# Recognition of environmental contaminant and pathogenic bacteria by means of redox potential methodology

**DOI:** 10.1016/j.mex.2024.102811

**Published:** 2024-06-19

**Authors:** Eya Yakdhane, Dóra Tőzsér, Oktay Haykir, Asma Yakdhane, Sabrine Labidi, Gabriella Kiskó, László Baranyai

**Affiliations:** a Institute of Food Science and Technology, Hungarian University of Agriculture and Life Sciences (MATE), Budapest 1118, Hungary; b Department of Food Hygiene, University of Veterinary Medicine, H-1078 Budapest, István u. 2., Hungary

**Keywords:** Multivariate classification, *Salmonella*, *Escherichia*, *Pseudomonas*, *Listeria*, Microbial growth model, Classification of bacterial strains based on redox potential curves

## Abstract

The time-consuming nature of culturing methods has urged the exploration of rapid modern technologies. One promising alternative utilizes redox potential, which describes the oxidative changes within complex media, indicating oxygen and nutrient consumption, as well as the production of reduced substances in the investigated biological system. Redox potential measurement can detect microbial activity within 16 h, what is significantly faster than the minimum 24 h incubation time of the reference plate counting technique. The redox potential based method can be specific with selective media, but bacterial strains have unique kinetic pattern as well. The proposed method suggests evaluation of the curve shape for the differentiation of environmental contaminant and pathogenic microbial strains. Six bacterial species were used in validation (*Escherichia coli*, Pseudomonas aeruginosa, Salmonella enterica, Listeria innocua, Listeria monocytogenes, and *Listeria ivanovii*). Descriptive parameters reached 98.2 % accuracy and Gompertz model achieved 91.6 % accuracy in classification of the selected 6 bacteria species.

• Mathematical model (Gompertz function) and first order descriptive parameters are suggested to describe the specific shape of redox potential curves, while Support Vector Machine (SVM) is recommended for classification.
• Due to the concentration dependent time to detection (TTD), pre-processing applies standardization according to the inflection point time.

## Graphical abstract

**Figure:**
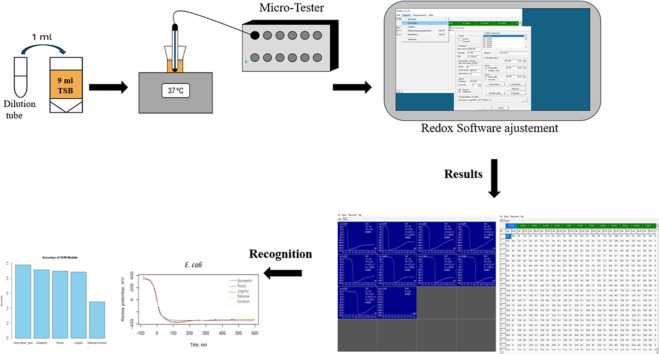

Specifications table

**Table:** 

Subject area:	Food Science
More specific subject area:	Food microbiology
Name of your method:	Classification of bacterial strains based on redox potential curves
Name and reference of original method:	Method of detecting and counting micro-organism in solid, liquid or aeriform substances. Hungarian Patent No. P0500591 https://www.sztnh.gov.hu/en Reichart et al. [9] Redox potential measurement as a rapid method for microbiological testing and its validation for coliform determination International Journal of Food Microbiology, Volume 114, Issue 2, Pages 143–148 10.1016/j.ijfoodmicro.2006.08.016
Resource availability:	R studio (version 2022.02.2, Build 485, Posit Software PBC, Boston, MA, USA) R (version 4.2.0, R Foundation for Statistical Computing, Vienna, Austria)

## Background

Ensuring the hygiene and safety of food products for consumers is vital, involving vigilant assessment of pathogenic microbes that present risks to human health. Traditionally, the identification of microorganisms is done by isolation of pure cultures pursued by physiological and biochemical tests. These methods are labour-intensive and time-consuming directing researchers to investigate alternative methodologies such as the flow cytometry method that utilizes cell penetrating fluorogenic substrates to Identify and count bacterial cells based on the light scattering properties. Despite its interesting results, this latter method faces the problem of instrument complexity requiring precise and specialized trainings [13].

Molecular methods provide a rapid and accurate results for identifying specific bacterial genes in biological solutions. However, they encounter a major disadvantage which is the elevated cost of materials and equipment used [11].

Moreover, various mathematical models are applied to predict and understand the microbial growth using influencing environmental parameters [1,8]. These models are basically used to monitor bacterial growth and are rarely used for bacterial identification.

An interesting innovative indirect method has been recently developed for microbial quantification. This method implies monitoring the microbial redox potential that serves as a fundamental physicochemical parameter, indicating the oxidizing or reducing capacity of biological systems. The negative redox potential value identifies a highly reducing environment, which is more likely to release electrons. Conversely, the positive redox potential value indicates an oxidizing environment that tends to accept electrons. When two redox systems come into contact, electrons generally move from the system of lower redox potential value to the one with the higher redox potential value, indicating the direction of electron transfer [6]. As a result of electron transfer, voltage can be measured, typically in the mV range. This parameter can be a significant indicator of microbial behaviour in their environment, since a reduction in redox potential indicates the proliferation of microorganisms in the measured environment. The extent and speed of this decline depends on factors like the growth rate, the oxygen and nutrient consumption and the physiological characteristics of microorganisms [9].

This redox potential method is cost-effective and simple to execute using a sensitive voltmeter device connected to measuring electrodes [12]. By analysing redox curves, a Time To Detection (TTD) value is derived, correlating with the initial cell count through linear regression [2]. Although the use of this method is limited to microbial quantification purposes, this approach can also be used to classify microorganisms.

This investigation focuses on validating the characterisation and classification of microorganisms using the redox potential curves of six bacterial species: *Escherichia coli*, Pseudomonas aeruginosa, Salmonella enterica, Listeria innocua, Listeria monocytogenes, and *Listeria ivanovii*. The curves were analysed using non-linear least squares regression and Support Vector Machine (SVM) for classification.

## Method details

The classification of bacterial strains using redox potential method involves four main steps:

### Preparation of bacterial strains

In order to collect appropriate quality redox potential curves, bacterial strains must be activated in suitable growth media and incubated at its optimal temperature for 24 h. The number of initial cells must be around 10^6^–10^7^ CFU ml^-1^. The dilution series should then be established and ready for the next step.

### Redox potential measurements and curves collection

The sample of 1 ml of each bacterial dilution tube must be pipetted into a sterile test cell containing 9 ml of half concentrated Trypto-Casein Soy broth. The electrodes are immersed inside the test cells after being disinfected with a 70 % ethanol solution. To cover the test cell, it is advised to use a silicone closure [9]. The tubes are immersed into a water bath to maintain a constant and optimal temperature for the bacteria being studied throughout the duration of the experiment. The initial redox potential value should be between 200 and 500 mV.

Measurements reading can be done manually or automatically using specific software. This includes the management of the start time, sampling time, and end of the measurements.

The sampling time is recommended as 10 min, and the end time is expected 10 h after the inflection point time.

The Time To Detection (TTD) value is the time when the absolute value of the redox potential change rate in the measuring test cell exceeds a value called Detection Criterion value (DC) which is described as the threshold value for the first derivative of redox potential data that is notably different from the random changes while the redox potential decreases. The DC parameter varies from bacterial species to another, but its absolute value is mostly ≥ 0.5 mV.min^–1^ [9].

**Fig. 1:**
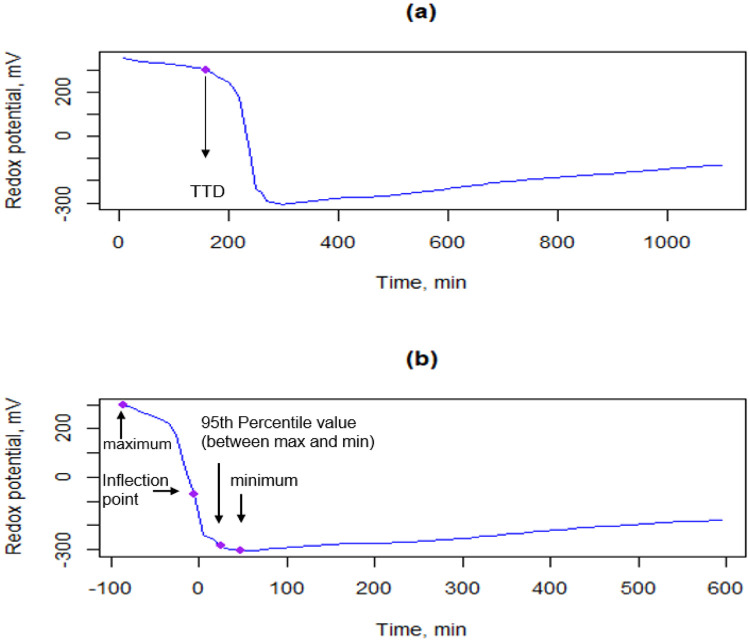
*E. coli* redox potential curves, (a) Acquired data with time to detection, (b) Normalized curve by adjusting the inflection time to zero.

The results of the redox potential measurements as a function of time can be then visualised by a curve (Fig. 1). Only curves with TTD should be selected for further analysis since they represent microbial activity and only these curves provide all the necessary parameters for curve fitting and classification process.

### Curve fitting and descriptive first-order statistical parameters determination

In this step the curves are normalized by adjusting the inflection time to zero since the TTD parameter is a concentration dependant and it increases as the bacterial concentration decreases. The descriptive first-order statistical parameters are determined to serve as reference in the determination of the model features or serve as classification parameters. These parameters include the maximum, minimum, inflection point, inflection point time, time corresponding to 95th percentile value between the maximum and minimum, and the slope of the final part beyond 95th percentile value.

For curve fitting, the Gompertz growth model was selected since it is the most accurate model from a total of four analysed growth models. Logistic and Rosso models, as well as a rational function were compared. The models are described in Table 1. The logistic, Gompertz, and Rosso growth models were selected because of their ability to provide meaningful interpretation within the biological context of their features. Additionally, the rational function was selected as a trial. Non-linear least squares procedure was performed to fit the Gompertz model on acquired data.

**Table 1:** Mathematical growth models for curve fitting on redox potential data.

Model	Equation	Parameters
Gompertz [14]	$R=\;A-Be^{-e^{-C(x-D)}}$	*A* = maximum value (mV) *B* = range between the maximum and the minimum values (mV) C = sharpness of the model *D* = normalized inflection point time (min)
Rosso [10]	$R=A-\frac{B}{1+\left(\;\frac{B}{C}\;\;\;-1\;\right)\;e^{-D(x-E)}\;}$	*A* = maximum value (mV) *B* = range between the maximum and the minimum values (mV) C = sharpness of the model *D* = absolute value of the detection criterion value(mV.min^-1^) *E* = normalized inflection point time (min)
Logistic [14]	$R=A-\frac{B}{1+e^{\left(\frac{4C}{B}\;\;\left(D-x\right)+2\right)}}$	*A* = maximum value (mV) *B* = range between the maximum and the minimum values (mV) C = sharpness of the model *D* = normalized inflection point time (min)
Rational function	$R=\frac{a_{0}+a_{1}x+a_{2}x2+a_{3}x3}{1+b_{1}x+b_{2}x2+b_{3}x3}$	a_0_ = maximum value (mV) a_1_ = minimum value (mV) a_2_, a_3_, b_1_, b_2_, b_3_ = coefficients without biological meaning

### Support vector machine analysis (SVM)

SVM is a supervised learning approach generally applied for classification and regression purposes [7]. This method was used to classify the bacterial strains. The classification can be established either using the fitted Gompertz model parameters or using directly the descriptive first-order statistical parameters. The kernel type for both cases should be radial kernel with default gamma value (1/ parameters number).

## Method validation

R studio (version 2022.02.2, Build 485, Posit Software PBC, Boston, MA, USA) and R (version 4.2.0, R Foundation for Statistical Computing, Vienna, Austria) were used to perform the non-linear curve fitting, and the Support Vector Machin (SVM) classification, as well as the bootstrapping for cross-validation.

**Fig. 2:**
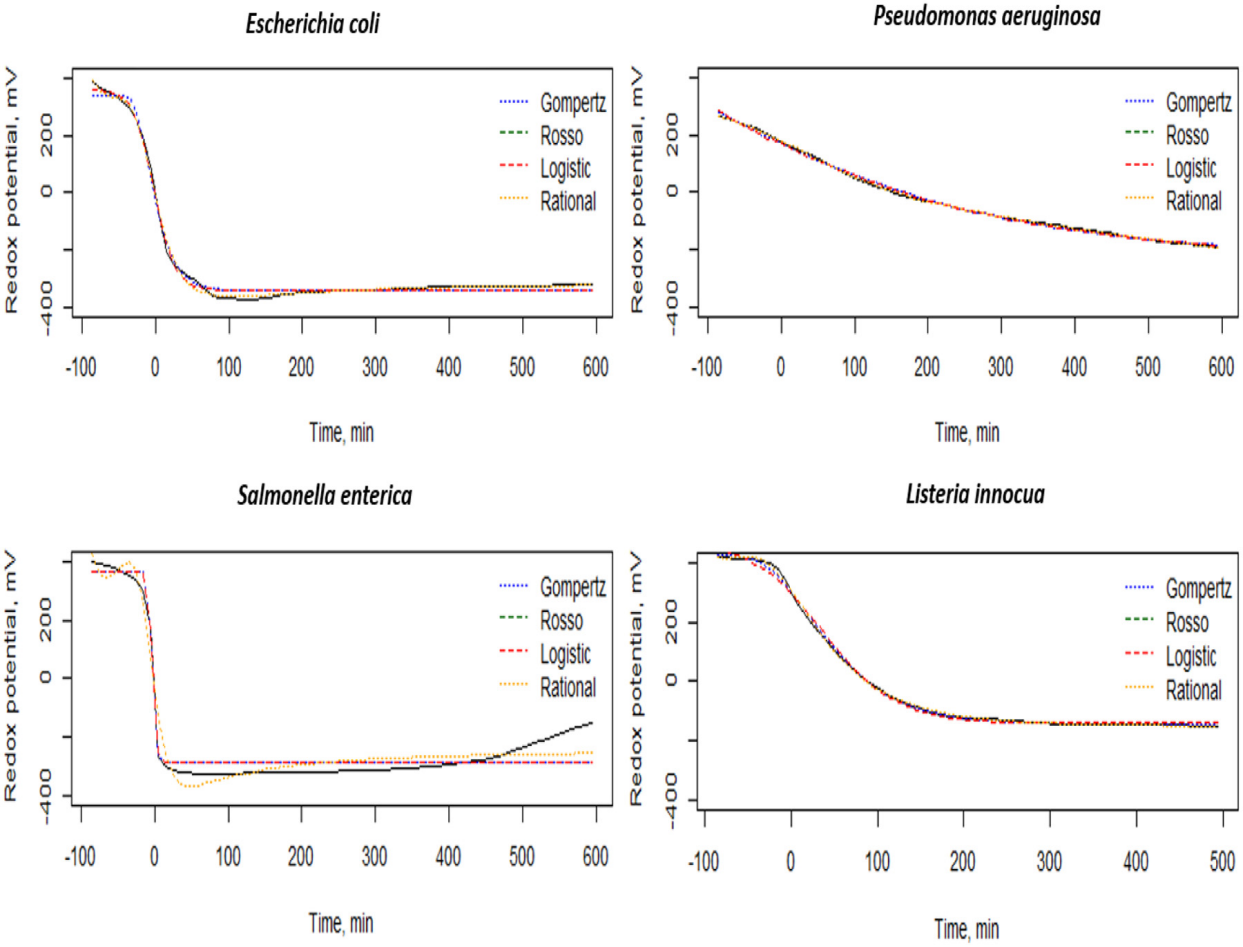
Example redox potential curves of *E. coli*, Pseudomonas aeruginosa, Salmonella enterica, and *Listeria innocua* with their fitted Gompertz, Rosso, Logistic, and Rational models.

To validate this method, *E. coli*, Salmonella enterica, Pseudomonas aeruginosa, *Listeria innocua, Listeria ivanovii*, and *Listeria monocytogenes* were investigated. These strains were obtained from the collection of the Department of Food Microbiology, Hygiene and Safety at the Hungarian University of Agriculture and Life Sciences. Strains were inoculated in a Trypto-Casein Soy Agar (TSA, Biokar, France) and incubated at 37 °C for 24 h in order to obtain a cell concentration around 7 log_10_ CFU ml^-1^. A tenfold serial dilution was then established till 10^–7^ as a preparation for the redox measurements [3]. 1 ml of each dilution was added to 9 ml of double-diluted Trypto-Casein Soy broth (TSB, Biokar, France). The redox potential measurements were carried out as previously described using the MicroTester device and Redox software (MicroTest Ltd., Budapest, Hungary). The results for each bacterial dilution obtained can be then illustrated in curves as it is presented in Fig. 1. (a). A total of 165 curves were collected. The obtained curve shape of *E. coli, Listeria ivanovii, Listeria innocua* and *Listeria monocytogenes* were remarkably similar to those illustrated in the studies of Szakmár et al. [12] and Erdősi et al. [4]. This observation underlines and supports the aim of our study to classify bacterial strains by their redox potential curves (Fig. 2).

After collecting all the descriptive parameters and the models features in a summarised table, bootstrapping was established for resampling and creating a larger dataset in order to enhance the robustness and efficiency of the classification analysis. The process implicated the creation of a larger dataset of 500 samples through random sampling with replacement from the original dataset and maintaining the same distributional properties. This approach allowed us to create diverse and balanced dataset that is suitable for the classification analysis and its validation.

**Fig. 3:**
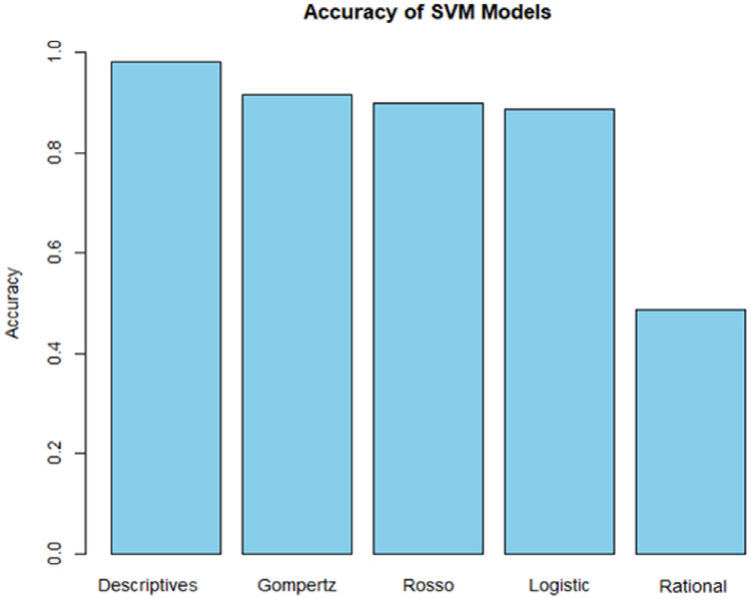
Accuracy comparison.

The classification accuracy of the studied models and the descriptive parameters is shown in Fig. 3. The analysis by SVM has shown that the differentiation of bacteria based on the descriptive parameters achieved the highest classification rate of 98.2 %. The confusion matrix (Table 2) showed that the majority of the classes were correctly classified.

**Table 2:** Confusion matrix of descriptive parameters in validation.

Recognition (%)						
	*Salmonella*	*Pseudomonas*	*E. coli*	*Listeria monocytogenes*	*Listeria ivanovii*	*Listeria innocua*
*Salmonella*	100	0	0	0	0	0
*Pseudomonas*	0	100	0	0	0	0
*E. coli*	0	0	100	0	0	0
*Listeria monocytogenes*	0	0	0	89.7	0	10.3
*Listeria ivanovii*	0	0	0	0	95.6	4.4
*Listeria innocua*	0	0	0	0	0	100
Overall accuracy = 98.2 %						

Approximately 10 % of the instances of *Listeria monocytogenes* and 4.4 % of *Listeria ivanovii* class were misclassified and merged with *Listeria innocua*. This misclassification is probably the result of the biological and biochemical similarities these three strains share, making their differentiation hard even with other techniques such as the international standard tests [5] that states that the existence of *Listeria monocytogenes* can be unnoticed in case of coexistence with *Listeria innocua* and *Listeria ivanovii*. This assumption was also mentioned in the study by Erdősi et al. [4], where while checking the presence of *Listeria monocytogenes* in raw milk and soft cheese by redox potential method they observed that this technique provided some false positive results in the presence of *Listeria innocua*.

Nevertheless, despite these misclassification levels, the results obtained from this model still indicate that the descriptive parameters were sufficient to achieve a nearly perfect classification among the studied bacteria.

The Gompertz model also achieved reliable results, with the classification accuracy of 91.6 % in validation (Table 3). For this model as well, some instances of *Listeria monocytogenes* and *Listeria ivanovii* were clustered with the class of *Listeria innocua*, resulting in strain specific misclassification rates of 47.4 % and 13.1 %, respectively.

**Table 3:** Confusion matrix of Gompertz model in validation.

Recognition (%)						
	*Salmonella*	*Pseudomonas*	*E. coli*	*Listeria monocytogenes*	*Listeria ivanovii*	*Listeria innocua*
*Salmonella*	100	0	0	0	0	0
*Pseudomonas*	0	100	0	0	0	0
*E. coli*	4.2	0	95.8	0	0	0
*Listeria monocytogenes*	0	0	0	52.6	0	47.4
*Listeria ivanovii*	0	0	0	0	86.9	13.1
*Listeria innocua*	0	0	0	0	1.3	98.7
Overall accuracy = 91.6 %						

Rosso, and Logistic models achieved respectable and comparable accuracy levels of 90 %, and 88 %, respectively. These two models obtained considerable misclassification of *Listeria monocytogenes* classes with the rate of 61 % and 62 %, respectively, as they were also clustered with *Listeria innocua*.

Compared to the other models, the rational function has shown the least accurate classification rate of 48.7 %. Its confusion matrix revealed that in almost all cases of mistake the bacteria species were recognized as *Listeria innocua*.

In conclusion, the statistical analysis has shown that the redox potential curves could potentially be distinguished. The descriptive parameters and the Gompertz model achieved a highest classification rate, making them the most appropriate models for the classification of environmental contaminant and pathogenic bacteria by means of redox potential curves. Presented results reached sufficient efficiency to suggest redox potential measurements for bacterial differentiation. This methodology has high potential for computer assisted detection of specific microorganisms and facilitating the assessment of measurement results.

## Limitations

The maximum determination level is 7 log units (10^7^ CFU/ml), because beyond this level the TTD is too short to be detected. Additionally, redox potential measurements are temperature sensitive, therefore accurate temperature control (±0.2 °C) is required for the water bath. Moreover, the production of gases by certain bacteria can cause fluctuations in the data which can disrupt the regularity of the recorded curves. This phenomenon is characterized by a noise what was mainly observed in case of *Salmonella*.

## CRediT authorship contribution statement

**Eya Yakdhane:** Conceptualization, Methodology, Validation, Writing – review & editing. **Dóra Tőzsér:** Methodology, Resources. **Oktay Haykir:** Investigation. **Asma Yakdhane:** Writing – original draft. **Sabrine Labidi:** Resources. **Gabriella Kiskó:** Supervision, Conceptualization, Writing – review & editing. **László Baranyai:** Supervision, Conceptualization, Investigation, Validation, Writing – review & editing.

## Declaration of competing interest

The authors declare that they have no known competing financial interests or personal relationships that could have appeared to influence the work reported in this paper.

## Data Availability

Data will be made available on request.
